# Syntax

**DOI:** 10.1002/wcs.1332

**Published:** 2014-12-10

**Authors:** David Adger

**Affiliations:** Queen Mary, University of LondonLondon, UK

## Abstract

Syntax is the cognitive capacity of human beings that allows us to connect linguistic meaning with linguistic form. The study of syntax is a huge field that has generated a great deal of empirical and theoretical work over the decades. This article outlines why understanding our syntactic capacity is important to cognitive science in general and why the data of syntactic research is to be taken seriously. It then provides an overview of a number of broad findings about the character of the syntax of human language, including evidence for abstract constituent structure, core properties of constituents, the importance of functional categories, the link between syntactic structure and meaning, and the range of types of syntactic dependencies, including dependencies of form, dependencies of position, and dependencies that create new meanings. *WIREs Cogn Sci* 2015, 6:131–147. doi: 10.1002/wcs.1332

## INTRODUCTION

Syntax is the cognitive capacity of human beings that allows us to connect linguistic meaning with linguistic form. The study of syntax has generated a great deal of empirical and theoretical knowledge over the decades.^1^–^4^ Here, I outline why understanding our syntactic capacity is important to cognitive science, and why the data of syntactic research is to be taken seriously. I then turn to a necessarily brief discussion of results that have emerged from syntactic work over the last half century or so. A striking fact, perhaps not obvious to those outside the field, is that there is a great deal of consensus as to the core problems, generalizations and range of solutions, even across widely differing syntactic theories.

I focus on syntax here, not language in general. Researchers outside of linguistics typically take the term language to have a common-sense meaning, connecting it to its functions in communication, sociality, creativity, cultural transmission, thinking etc. While these functions are fascinating and important, it is possible to investigate the syntax of language while leaving questions of its functions aside. Without an understanding of the nature of the capacity, questions as to how (or whether) its function has shaped that capacity are shots in the dark.

## WHY IS SYNTACTIC THEORY RELEVANT TO COGNITIVE SCIENCE?

Sentences (and other expressions) of human languages have structure relevant to their meaning and pronunciation, and syntactic research attempts to uncover that structure. Understanding the results of that research is therefore key to understanding language as a whole. This structure is why *Anson bit Lilly* does not mean the same as *Lilly bit Anson*, even though both expressions consist of the same words. It is also why the words *her* and *she* differ in pronunciation, even though they refer to the same individual and play a close to identical semantic role, in sentences like *Anson believes her to be vicious* and *Anson believes that she is vicious*.

One might think that, in the cases just mentioned, what is relevant is merely the order of the words, which might be sufficient to determine both meaning and pronunciation. To see that order will not suffice, compare *Anson bit Lilly* with *Lilly was bitten*. Here, *Lilly* precedes rather than follows *bite*, but it has the same meaning with respect to *bite* as it does in *Anson bit Lilly*: in both cases, Lilly suffers the bite. At a slightly more abstract level, take the sentences *Lilly fears Jasper* and *Lilly amuses Jasper*. In the first, the individual who is feeling the emotion is denoted by the word that precedes the verb, while in the latter, the individual feeling the emotion follows the verb. Such effects, where meaning is divorced from word order, are ubiquitous in language.

It would be trivial to define an artificial language that defines semantic roles on the basis of order (like, for example, standard translations of English into predicate logic do). A system like that would just match cognitive semantic concepts relevant to linguistic meaning to surface properties of the ordering of words; however, no human language works like this.

Another intuitively simple system would be one where all the grammatical relationships between words are confined to words that are next to each other. However, human languages do not work like that either.

For example, in the general case, the form of a verb is not determined by the adjacent words. This is why in *The girls from Paris are singing*, the auxiliary verb form of *be* appears as the plural version *are* and not the singular *is*, even though *are* is directly adjacent to the singular noun *Paris* but distant from the plural noun *girls*. Compare this with *Paris is beautiful*, which shows that *Paris* can trigger a singular form of the verb. The examples differ because *Paris* is in a different structural relationship to the verb in the two cases, even though it is adjacent to the verb in both.

Such simple examples are important because they are general across human languages: no language that we know of has a general rule that will trigger verbal agreement in number with the equivalent of *Paris* in *The girls from Paris are singing*; simple contiguity is never the precondition for this kind of verb agreement. Even more interesting is that speech errors often give rise to such agreement (‘attraction errors’^5^), but these are never generalized by children learning the language to a rule that causes the verb to agree with the adjacent noun. If they were so generalized then the result would be a paradigm that looks as follows, with the reverse of the usual English pattern in (a–d) but the standard English pattern in (e–h) (I present data from now on numbered as is standard in linguistics, with a * to signify an empirical claim that the sentence is not acceptable in the language in question. *The Question of Data* discusses the nature of syntactic data in more depth):

(1) **a**. The girls from Paris is singing
**b**. *The girls from Paris are singing
**c.** The girl from the Western Isles are singing
d.*The girl from the Western Isles is singing
**e.** Paris is beautiful
**f.** *Paris are beautiful
**g.** *The Western Isles is beautiful
**h.** The Western Isles are beautiful

Not only does no variety of English work like this, no variety of any human language works like this.

From the perspective of cognitive science, such observations are fundamental: they show that the most obvious surface properties of words (their order and their contiguity) are not properties that are important in how human language negotiates the relationship between form and meaning. If order and contiguity were the organizing principles of language, then, given that agreement is particularly vulnerable in language change,^6^ and attraction errors exist,^5^ languages should drift toward an adjacency based agreement system. However, they do not. Rather human language learners ignore the adjacency relationship as a potential hypothesis and successfully learn that even distant nouns can determine the verb form:

(2) The **girls** from Paris that the cat scratched in the house **are** singing.

This entails that human language learners are biased towards structural conditions on grammatical dependencies and against surface conditions^7^–^9^.

The result that structural not surface properties are fundamental to human language, stable since the 1950s in linguistics, has a great deal of psychological and neuropsychological confirmation. Neuroscientfic investigation has consistently found that there are different patterns of brain activation when humans learn artificial languages which are based on structure versus surface order.^10^

## THE QUESTION OF DATA

Psychologists and syntacticians work on very different kinds of data. For the most part, the data that syntacticians work with will have medium to large effect sizes (Cohen's *d*^11^): that is, the mean difference in a measured behavioral reaction to a stimulus divided by the mean standard deviation is much greater than 0.5. Effect size is a measure of the strength of a phenomenon and Cohen defines medium to large effect sizes as those that a trained researcher can see without applying statistical analysis.^12^ Sprouse and Almeida^12^ show that, for a large and representative sample of syntactic data (collected via traditional, informal methods), formal experimental investigation converges with the informally collected data (97% of the relevant phenomena covered in a graduate textbook^13^ and 95% of the English data in a decade of Linguistic Inquiry articles). They attribute this in part to the fact that the data used to make scientific claims about the syntax of human languages, in general, have medium to large effect sizes.

To get a sense of the issues, take a simple set of sentences like the following:

(3) **a.** I met someone from New York at the conference.
**b.** From New York I met someone at the conference.
**c.** At the conference I met someone from New York.

The empirical task the syntactician sets him/herself is to determine whether (b) or (c) is most closely related to (a). Without performing any statistical test, it's clear that (c) is related to (a) more closely than (b) is, and, in fact, (b) is not acceptable as a sentence of English with the same meaning as (a), while (c) is. This is a typical datum in syntax, and in fact it can be used as an argument in a fairly sophisticated chain of reasoning about what the structure of (a) is. The strength of the effect size is evident to any speaker of English.

This data is exactly the kind of data that most syntacticians work with on a daily basis, and the strength of the effect is so clear that most syntacticians feel justified in not subjecting it to statistical testing,^12^–^15^ although this view is not universal.^16^ Further, the speed with which this kind of data can be amassed has allowed syntacticians to build a large and solid empirical understanding of syntax across many languages. For example, translating the sentences above into other languages, perhaps languages with quite different grammatical properties from English, can quickly lead to an understanding of how consistent or variable the phenomenon is, and to what extent it correlates with various other properties.

Does this mean that syntacticians should not perform experiments to test the strength of their claims? Certainly not. In some cases the effect size is not clear to the researcher. This can be tackled via more careful design of the materials to weed out pragmatic or semantic factors that interfere but sometimes data will simply not be considered to have a strong enough effect size to be used in theoretical or analytical argumentation. Experimental approaches are sometimes necessary.^17^–^19^ Similar comments are true for corpus data, which have advantages in providing frequency information, but disadvantages in the rarity of the crucial examples.^14^

The lack of statistical and other information about data provenance and analysis does raise a communication difficulty: psychologists looking at syntax papers and seeking the kind of statistical information they are used to in their own discipline will generally find it lacking. However, the field itself clearly has mechanisms for ensuring the solidity of the data that it uses to build theoretical claims on, and as Sprouse and Almeida's work shows, the vast majority of the data used in theoretical argumentation are replicable and reliable, because the size of the effect is immediately and easily checked. Different disciplines have different methods of establishing their core phenomena. Judgments of acceptability applied to well designed materials in syntax have been crucial in building a very rich base of cross-linguistically valid knowledge about syntactic structure.

## IMPORTANT FINDINGS IN SYNTAX

In *Why Is Syntactic Theory Relevant to Cognitive Science?* we saw that structure, not surface order or contiguity, is relevant to how meaning and form are related in human language. An equally important empirical point about the meaning-form relationship is that it is astoundingly vast: a speaker of a language, who has acquired a finite set of words in that language, can link the forms of sentences in the language to their meanings over a range of experiences so large that it seems senseless to place an arbitrary limit on it. Cortical and psychological evidence suggests that learning the grammar of a language is complete by puberty.^20^,^21^ It follows that speakers acquire a productive means to link form and meaning (a grammar) over a practically unbounded range, from a bounded set of experiences. The question is how to model such a capacity.

Perhaps the major insight of generative syntax is that a human grammar can be modeled by a finite mathematical function. This function, in the early years of investigation, was taken to be fairly complex, modeling a number of levels of linguistic description, and leading to a characterization of human linguistic knowledge that seemed highly particular.^22^–^24^ The general framework, in its many incarnations, allowed an explosive growth in empirical knowledge about the syntax of languages as well as increasing depth of analysis and a series of well understood high-level generalizations about phenomena. More recent models have radically reduced in complexity as the empirical generalizations have become clearer,^25^ allowing far less innate structure to be imputed to the linguistic system.^26^

We can divide the high level generalizations discovered over the years into two broad classes: generalizations to do with the *shape* and *meanings* of structures and generalizations about *dependencies* between elements within these structures.

### The Shape of Grammar: Constituency

Constituent structure is the grouping of linguistic elements together to the exclusion of other elements, establishing another dimension of organization than the surface order of the elements in a sentence. Usually this is represented as either a bracketting, or a tree-like structure:

(4) a. [ A [ [ B C ] D ] ]

b.
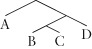


The expressions grouped into a single constituent are said to be *sisters*, while the overarching constituent that contains them is termed the *mother*. Typically, it is assumed that there is information specified at the juncture points in the tree as well as at the terminal nodes of the tree. This means that these nodes are *labeled* in some fashion. For example:

(5) [_A′_ A [_D′_ [_B′_ B C ] D ] ]

b. 
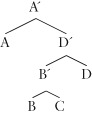


Labeling is distinct from constituency, and indeed some approaches in syntax eschew it,^27^ preferring to specify all the relevant information in the terminal nodes.

#### Evidence for Constituent Structure

(i) Constituents provide a description of distributional patterns in languages that allows linguists to capture a wide range of facts about particular languages via a condensed set of constituent structure types; this is what allows us to capture the fact that a pronoun like *she* and a phrase like *David and Anson's sleepy cat* are intersubstitutable in English in a wide range of syntactic contexts (they are all characterizable as a constituent with the same kind of node label), while at the same time capturing the fact that there is no word that is intersubstitutable with, say, *and Anson's sleepy* which is a subsequence of *David and Anson's sleepy cat* but not a constituent of it. Such distributional generalizations were the initial motivation for constituent structure.^28^

(ii) Many overarching syntactic generalizations that govern sentence structure are typically statable in terms of constituents. For example, in many Germanic languages, such as Swedish, the verb (bolded in (6)) in a main clause must be preceded by exactly one constituent (Holmberg^29^ example 1):^a^

(6) 
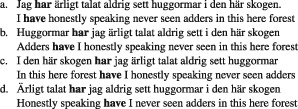


The cases in (6) are representative examples of a phenomenon which is best characterized by requiring that the tense marking auxiliary verb (**have**) appears after the first constituent of the sentence.

More generally, a wide range of syntactic generalizations are sensitive to constituents (see any syntax textbook for a discussion^13^,^30^,^31^). For example, the agreement phenomenon mentioned in *Why Is Syntactic Theory Relevant to Cognitive Science?* is captured by saying that the verb agrees with the whole constituent *the girls from Paris* rather than the adjacent noun *Paris*. Such generalizations are established within languages and typically converge on identical assignments of constituent structure (though there are cases of interesting mismatches^32^–^34^), and the methods for determining constituency are applicable across languages.

(iii) Constituents display both semantic and phonological unity to the extent that theories of sentence semantics^35^ and phonology^36^ make crucial use of them. Relatedly, assigning independently motivated constituent structures correctly predicts a range of semantic ambiguities without the need for special stipulations. For example, it is possible to modify a noun with an adjective as in *sleepy cats* and it is also possible to conjoin two nouns into a single constituent as in *cats and dogs*. It follows with no further stipulation that the phrase *sleepy cats and dogs* will be ambiguous, since that sequence of words will be consistent with two assignments of constituent structure (I assume a binary constituency here, although that is not relevant to the argument):

(7)
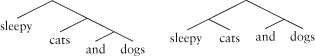


In the structure on the left the adjective *sleepy* modifies the whole structure *cats and dogs* while in the tree on the right it modifies just *cats*, capturing the ambiguity and the natural position to place pauses if a speaker wishes to prosodically disambiguate the phrase. These kinds of phenomena are pervasive and constituency provides an elegant way of capturing them.

(iv) Psychological evidence abounds that these constituent structures (or at least some of them) are relevant in sentence processing: the structural priming phenomenon,^37^,^38^ perception experiments,^39^–^41^ relatedness judgments,^42^ slips of the tongue effects^43^ etc.

#### Properties of Constituent Structure

(i) Current views of syntax take the number of subunits of a constituent to be severely limited. The standard position within the Minimalist framework^44^ is that constituents are uniformly binary (a position that is held also within Categorial Grammar approaches), but other frameworks also have severely restricted branching (although some take structural complexity to reside in conceptual structure, rather than in syntax^45^). The fundamental empirical reason for this is that models of the data that incorporate highly layered structures are empirically more successful than those that do not. For example, in English, it is possible to elide constituents containing the verb, as can be seen from the grammaticality of the sentences in (8). This is easily captured if the structure is roughly that in (9), and the generalization about elision is that you can elide constituents:

(8) **a.** Lilly might have been running.
**b.** Lilly might have been.
**c.** Lilly might have.
**d.** Lilly might.

(9) [ Lilly [ might [ have [ been [ running ] ] ] ] ]

(ii) It is also a close to consensus position that word order is to be factored out from a specification of hierarchical structure.^46^,^47^ That is, constituent structure is independent of the order of the elements in that structure, so the two trees in (10) are equivalent ways of representing the same constituent:

(10)
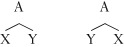


The strongest argument for this comes from the elegant analysis it gives of aspects of cross-linguistic variation. Consider the English sentence:

(11) Anson said Lilly bit Michael.

In Japanese, this translates to the equivalent of (12):

(12) Anson-wa Lilly-ga Michael-o kanda-to itta.

Anson Lilly Michael bit said ‘Anson said Lilly bit Michael.’

For both languages, the evidence is that the constituency is identical.^13^,^48^ We thus have the following constituent trees where the structure is exactly the same, but the order is reversed under the nodes F and G:

(13)
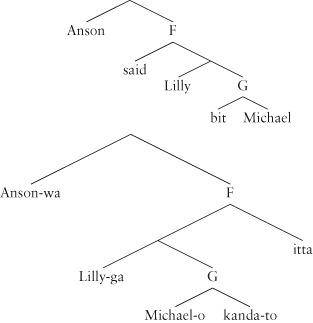


Even those languages which have been claimed to have extremely free word order (for example the Pama-Nguyan language Warlpiri or the Kiowa-Tanoan language Kiowa), with no obvious evidence for constituent structure of the sort discussed here, have turned out not to involve random permutations on a very flat structure. Contrary to the original proposal of Hale,^49^ detailed empirical work has shown that these languages have clear hierarchy effects, suggesting constituent structure.^50^,^51^ However the debate is still open as to how to model these empirical findings.

(iii) The third important consensus position on properties of constituent structure is the idea that the type of a constituent is predictable from the type of a constituent contained within it (in the simplest case a single word, or subpart of a word, called the *head*). Evidence for this is that, within a constituent, there is usually a single element which is of primary importance in determining the grammatical behavior of the whole unit. For example, in *the girls from Paris*, the word *girls* determines the agreement on the verb, and so is the head. Another kind of example is that the grammaticality or ungrammaticality of the phrase *ate the mouse* in a context is more or less identical to the grammaticality or ungrammaticality of the word *ate* in that context, so *ate* is the head.

#### Specifiers and Complements

It will be useful here to introduce a simple piece of technical terminology:

(14)**a.** If a phrasal constituent is the sister of a head, it is said to be the *complement* of that head
**b.** If a phrasal constituent is the sister of the mother of a head, it is said to be the *specifier* of that head

Simplifying somewhat, a phrasal constituent is just a constituent that has internal structure (i.e. has further constituents internal to it). In a simple tree, we can talk about the various sections of the tree with respect to the head as follows:

(15)
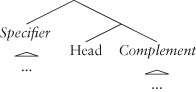


These two technical terms turn out to be useful in discussing important recurring structural relationships found in human languages.

#### Functional Categories in Constituent Structure

The proposal that all constituents have a head has led to an analysis of sentences as phrases headed by an item that marks the grammatical tense (whether the sentence is past, present, or non-tensed), often simply notated T, so that a simple (emphatic) sentence like *Lilly did jump* looks as follows, where T is (the label of) the head of the sentence and is pronounced as *did*. The specifier of T (that is, the sister of the mother of T) is the traditional subject of the sentence (*Lilly*, in this case), and T's complement is the constituent whose head is the verb *jump* (as is standard, I use a triangle in the tree to gloss over irrelevant structure; see *How Meaning Links to Structure—Verbs* for discussion of the verb phrase). Traditionally, the whole sentence is also taken to bear the label T, although other possibilities have been and are being explored.^8^

(16)
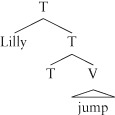


Categories like T are known as *functional categories*, and have become extremely important in modern syntactic research^52^:

(i) Functional categories are linguistically distinct from the categories that distinguish content words (usually called *lexical categories*, for example, *cat* N, *jump* V and *happy* A(djective)). The lexical categories N, V and A distinguish classes of content words, each class having many members. Functional categories label far fewer expressions (e.g. the (C)omplementizer category introduced below has only three members in English) which means that particular tokens of functional categories are frequent in contrast to tokens of lexical categories, with implications for language acquisition.^53^

(ii) There are a number of linguistic differences between functional categories and lexical categories. Phonologically, the expressions labeled by functional categories tend to be shorter, non-stress bearing and often have particular sets of phonological constraints imposed upon them (such as a restricted set of consonants or vowels.^54^ They are also often affixes, attaching to lexical categories (e.g. the affix -*ed* marking past tense attaching to the verb in *jumped*). Semantically, they specify grammatically relevant meanings such as tense, aspect, definiteness, plurality, etc. These are rigid, fixed points in meaning,^55^ specified using well-understood logical techniques. In contrast, content words like *jump* are semantically vague and resist complete analysis using logical techniques.

(iii) Expanding beyond these domains, functional categories and lexical categories are differently affected in language disorders,^56^ language acquisition,^57^ and neurophysiological response.^58^

The widespread acceptance of functional categories, plus the consensus on limited (perhaps binary) branching structure, can lead to a fair amount of complexity in syntactic representations: it is not untypical to see tree representations spanning whole pages of articles. However, this complexity should not be confused with abstractness. In a full-fledged analysis, each functional category has a meaning, so each is semantically concrete. Most have either direct phonological expression in the relevant syntactic position, or are in a syntactic dependency with another element that phonologically expresses the particular value of the category. For example, in the tree above, T marks the temporal semantics and is pronounced as *did*, but the overt expression of T can also appear as an inflection on the verb (for example -*ed*), possibly non-contiguous with T (as in *Lilly often jumped*).

The whole tense constituent (sometimes called TP, for Tense Phrase on analogy with VP, for Verb Phrase) is the sister of another functional category, the complementizer (C). C may be overtly expressed in English when a sentence combines with a verb:

(17)**a.** I know **that** Lilly did jump.
**b.** I asked **if** Lilly did jump.

Whether the embedded sentence *Lilly did jump* expresses a fact or asks a question is marked overtly in English by the distinction between the complementizers *if* and *that*.

Functional categories tend to be strictly hierarchically ordered with respect to each other. For example, taking *that* to be a C, and emphatic *did* to be T, we find only one order is possible, captured by requiring C to be hierarchically superior to T:

(18)**a.** I know that Lilly did jump.
**b.** *I know did Lilly that jump.

(19)
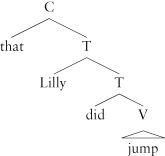


Evidence from distribution, semantics, constituency and order has been used to establish a number of functional categories that appear in both sentences and noun phrases in many different languages.^59^ For example, in the noun phrase in English, a demonstrative (Dem) like *those* occurs before a numeral (Num) like *three*, which in turn occurs before an adjective like *big*:

(20)**a.** Those three big oranges
**b.** *Big three those oranges

As we have seen, hierarchy is distinct from order, predicting the existence of languages that reverse the English order, while maintaining the hierarchy. Such languages are common; for example, the phrase translated into Thai would be:

(21)sôm jàj saam-lûuk nán
orange big three those

This finding can be modeled using a hierarchical structure as follows:

(22)
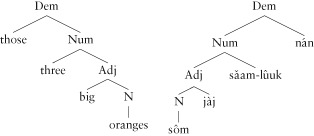


The lexical categories of noun and verb are surmounted by a (hierarchically) ordered sequence of functional categories carrying logical semantic information. Such sequences are termed Extended Projections.^60^ There is a great deal of debate about the richness, hierarchical order and universality of these functional categories, but there is a strong consensus that they are required.

### How Meaning Links to Structure—Verbs

We now turn to the meanings associated with lexical categories, restricting our attention to verbs. Verbs typically occur with noun phrases that are semantically connected to them: for example, a verb like *jump* has a single semantic participant, expressed by the noun phrase *the cat*, in *the cat jumped*. A number of important generalizations have been discovered about these verb noun phrase relations over the years. We will discuss just one: the noun phrase that denotes the agent of an event is hierarchically superior to the noun phrase that denotes a non-agent.^61^ In the example, *Anson bit Lilly*, the subject *Anson* is external to the constituent *bit Lilly*, hence hierarchically superior to *Lilly*, and it is interpreted as the agent of the event. This correlation between semantic roles and syntactic hierarchy is very strong, holding across verb classes and across languages, with apparent exceptions reducing to independent factors.

One way that this generalization has been embedded in theoretical models is to tie the meaning of Agent to a functional category that specifies that the subject is the Agent of an event denoted by the verb phrase. This functional category is hierarchically sandwiched between V and T in the extended projection. This proposal decomposes the verb into two syntactic components, the contentful lexical verb *bite* and a functional element notated v (pronounced ‘little v’). The semantic function of v is to add an agent to the verb and approaches have been developed which execute this in different ways^62^–^64^:

(23)
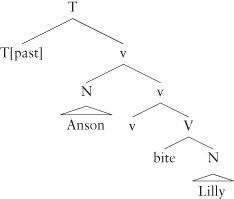


The meaning of v is specified so that *Anson* is interpreted as the agent of *bite Lilly*, while T marks this event as taking place before the speech time (as is common, I have notated the fact that we have a past tense here by writing [past] next to the category T). I return below to how the word order is captured. Empirical arguments have been given that in various languages v is phonologically expressed (e.g. the Mayan language, Chol^65^), although this is an ongoing area of investigation, and much is still unclear. An alternative to this view takes the verb itself to specify that the participant that is interpreted as the Agent is to be placed syntactically higher, so that the relevant generalization is one about the way that information specified of a word maps to syntactic information.^66^–^68^

Further work, following on from Dowty's early investigations,^69^ has connected the interpretation of the subject and the object to aspectual properties of the verb.^70^ The more general outcome of this research is that there is only a small number of basic event types and evidence that these correlate tightly with a small number of basic syntactic structural types.^71^

To get a flavor of this, consider the possible syntactic positions for noun phrases that denote participants of the event denoted by a verb (these are usually called the verb's *arguments*). If the verb has two arguments (traditionally, these are termed *transitive* verbs), as in (23) above, there is no ambiguity about which is which, given the meaning associated with v and its syntactic position relative to V. However, if a verb has just one argument (i.e. it is *intransitive*), then there are two available syntactic positions for that one argument: either where *Lilly* is placed in (23), or where *Anson* is placed. Further, each of these is associated, by hypothesis, with a meaning: the element that combines with the constituent headed by v is associated with an Agent meaning, while the sister of the verb is not. We predict two kinds of intransitive verb:

(24)
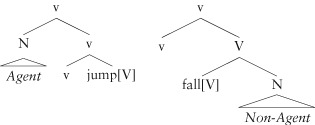


The two classes of intransitive verb in (24) (termed *unergative* and *unaccusative* verbs) have been empirically recognized since the 1970s^72^ and there is a wealth of linguistic evidence that they are structurally, and not just semantically, distinct. The unergative verb *jump*, which has an Agent, would have the representation on the left, while the unaccusative *fall*, which lacks an Agent, would have the structure on the right. In English, this structural difference is masked for independent reasons (see below), however, in other languages, numerous grammatical distinctions are dependent on this difference, including the kind of auxiliary that the verb appears with, the possible positions for the noun phrase, and the behavior of pronominal elements in clause structure (see the introduction to Ref 73 for review of the phenomena). There is also processing evidence for the distinction from different experimental paradigms.^74^–^76^

### Syntactic Dependencies

Expressions of human language have a constituent structure and there is evidence for the shape of that structure and for how it interacts with meaning. We now turn to the dependencies that hold between constituents. These dependencies are crucial for determining various aspects of both form and meaning. We will distinguish first between *form-related* and *position-related* dependencies. The first arises when one constituent influences the pronounced form of another, and the second when one element in structure requires another element to be in a certain position with respect to it. We then turn to *meaning-related* dependencies, where constituent structure constrains what meanings are available for relating constituents within a structure.

#### Form Related Dependencies

We have already encountered a form-related dependency in *Why Is Syntactic Theory Relevant to Cognitive Science?*—agreement. Agreement is a systematic covariation in the linguistic forms of two or more elements. However, care must be taken as to what we mean by ‘form’ here. In English examples like (25), it is not the formal plural marking on *girls* (i.e. the -*s*) that covaries with the form of the verb, but rather a more abstract property of this word, which is interpreted as semantic plurality. (25c) shows that the form of the word is not relevant (as the word ‘sheep’ does not change its form to mark whether it is singular or plural). It is also not obvious that it is the meaning that is conditioning the agreement, unless we take electronic scales to be semantically plural.^77^ In other languages, as we will see directly, appeal to meaning is not sufficient to explain agreement.

(25)**a.** The girls are singing.
**b.** *The girl are singing
**c.** The sheep is/are bleating.
**d.** The electronic scales are broken.

A grammatically relevant property of a word or other linguistic expression is called a (syntactic) *feature*: we say that the number feature on the noun (marking its semantics as singular or plural) covaries with a number feature on the verb, which is responsible for the form of the verb.

As well as subject verb agreement, languages also display agreement of the verb with objects. For example, the finite verb in the Nakh-Daghestanian language Tsez agrees, in the usual case, with the noun class of its object^b^

(26) eniy-ā ziya b-išer-si
mother cow fed
‘The mother fed the cow.’ (Polinsky and Potsdam^78^)

Cow in Tsez is a class III noun. Notice the *b*- at the start of the verb, and compare with (27), where the object is *girl*, a class II noun, and the verb is instead prefixed with *y*. This is evidence of an agreement dependency between the verb and its object sensitive to the class feature of the object.

(27) eniy-ā kid y-išer-si
mother girl.II II-fed
‘The mother fed the girl.’

Some languages allow both subject and object agreement, and in others, agreement can appear for three arguments of a verb.

Another important set of syntactic dependencies involving form are case-dependencies. Take the following sentences in English:

(28)**a.** He saw her.
**b.** *Him saw her
**c.** *She saw he
**d.** She saw him.

Here the form of the pronoun is apparently dependent on its syntactic position. The traditional notion of ‘case’ captures this: nouns and pronouns come in different shapes which are dependent on a case feature they bear, and this case feature is dependent on the syntax. In the examples above, the forms *he*, *she* are *nominative* while the forms *her*, *him* are *accusative*. It turns out that it is not possible to associate case features with meanings directly (as is shown by comparing *She bit me* versus *She was bitten*, where the same case (nominative) appears with different semantic roles (Agent and Non-Agent, respectively). At least some case phenomena are connected to structure not to semantics.

#### Position Related Dependencies

In addition to these dependencies of form, we also see dependencies of position, where one element (usually a head) requires the presence of another particular element in a structurally local position. One such dependency is *subcategorization* (often called *complement selection*). For example, a verb like *hit* requires an object: *Lilly hit the mouse* versus *Lilly hit. Moreover the object has to be a certain syntactic category (in this case a noun phrase: cf. *Lilly hit that Anson sneezed, or compare *I depend on you*/ *I depend you). Subcategorization dependencies are known to be psychologically very salient, to bear a close but indirect relationship to verb meanings,^79^ but to be not entirely reducible to meaning.^80^ To see this consider:

(29)**a.** I asked the time.
**b.** I asked what the time was.
**c.** *I inquired the time
**d.** I inquired what the time was.

Here the verbs ‘ask’ and ‘inquire’ both combine with a complement whose meaning is a question, giving (29b) and (29d). However, only ‘ask’ can combine with a noun phrase object which has a question meaning, so (29c) is semantically reasonable but lower in acceptability than the other examples. This pattern suggests that there is a role for purely syntactic information that specifies the kind of object a verb requires (noun phrase or sentence), presumably learned as a feature of the verb in addition to the verb's meaning.

In our discussion of unaccusative and unergative verb classes, we saw that there was good linguistic and processing evidence for two structural positions: unaccusative verbs' single argument is the sister of the verb, while unergatives' is higher in the structure, the specifier of v. This is a subcategorizational difference in verb class: unaccusatives like *fall*, which have no Agent, take their single argument as an object, unergatives like *jump* take their argument as the specifier of v. However, looking again at the trees in (24), one might expect the object in the unaccusative to occur after the verb, since verb–object is the general order in the English verb phrase; but this is not what we find. Subjects of both unergatives and unaccusatives precede the verb:

(30)**a.** The cat jumped.
**b.** The cat fell.

The reason for this is another positional dependency, but this time one that is purely syntactic: in English the specifier of T must be filled by some constituent. Syntacticians call this position the *structural subject* position. This generalization about English grammar is motivated by a simple observation: sentences in English never lack a structural subject:

(31)**a.** (I know that) a cat is in the lavender.
**b.** (I know that) there is a cat in the lavender.
**c.** *(I know that) is a cat in the lavender
**d.** It is 4 o'clock. /*is 4 o'clock

(31b) requires the ‘dummy’ word *there* to be in the position before the auxiliary verb *is* as we can see in (31c). Similarly, (31d) requires the pronoun *it*. The generalization can be stated as:

(32) The structural subject position (i.e. the specifier of T) must be filled in English.

This generalization does not hold in other languages (it is a point of syntactic variation) and whether it has a deeper explanation is a question of some controversy. There are also exceptions (for example, imperatives, like *leave now!*) although these are the result of well-understood interfering factors.

However, taking this generalization to be true for English, we now have an explanation for why the surface orders of unaccusatives and unergatives is the same in English. Even though the two verb phrases are distinct in structure, this difference is obliterated, because (32) requires the single noun phrase in each structure to appear in the structural subject position. There are, then, two selectional requirements here: one in the verb phrase where the interpretation of the noun phrase as an Agent or not is determined, and the other higher in the sentence, where requires there to be a structural subject. These two selectional requirements can both be met by taking the phrase *the cat* to be in both structural positions simultaneously, giving the following representations:

(33)
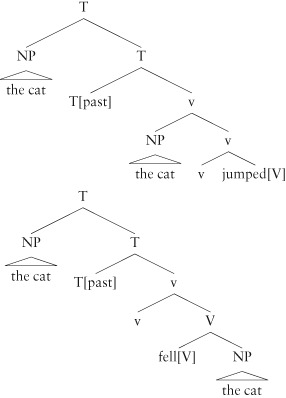


In English, the higher of the two *the cat*s is pronounced, so the surface order is the same. These simple examples have two types of positional dependency relations: a dependency between the verb and its argument, which has a semantic effect, and a distributional regularity of English that requires a structural subject, and hence has an effect on word order.

This phenomenon, where a single pronounced item enters into two (or more) dependencies in a structure is very common, and it is often termed ‘movement’ or ‘displacement’. The appropriate theoretical implementation of this phenomenon is controversial, but is orthogonal to its existence, and ubiquity, in languages of the world.

#### Meaning-Related Dependencies

An interesting meaning-related dependency which has a structural correlate involves what are called *bound variable* effects. The following example has two distinct meanings:

(34) Every kitten wanted its food.

The most salient meaning is that kitten A wants the food that belongs to kitten A, while kitten B wants the food that belongs to kitten B etc. This is the *bound variable reading*, as it can be represented in (Anglicized) predicate logic (35) as, where the variable *x* is bound by the quantifier *every*:

(35) For every x, where x is a kitten, x wants x's food.

The other meaning is that there is some other individual (say a puppy) whose food we are talking about:

(36)**a.** The puppy tore into the steak it had stolen. Every kitten wanted its food.
**b.** There is some y (a puppy) and for every x, where x is a kitten, x wants y's food.

When we change the structural relationship between the phrase *every kitten* and the pronoun *its*, the bound variable reading vanishes (or at least becomes much less accessible).

(37) Its food was in every kitten's bowl.

This phenomenon is highly complex, and structural conditions are possibly only part of what is going on,^81^ although the current consensus position is that the structural relation of *c-command*, a kind of extension of sisterhood, is implicated (see sidebar).

Another case where we see bound variable effects (and c-command) is *reflexives*, words like *herself* in English. Consider:

(38) Lilly impresses herself.

The meaning of this requires the two arguments of the verb *impress* to be construed as the same individual. That is, the verb phrase *impresses herself* means something like *x, such that x impresses x*, where we fill in the variable x with the meaning of the noun *Lilly*. We see a similar effect with the expression *each other*:

(39) Lilly and Jasper impressed each other

Here the verb phrase means something like *x and y, such that x impresses y and y impresses x*.

**C-command**

The syntactic relation mentioned in the text is called *c-command* (for constituent command) and can be defined as follows^13^:

(i) A constituent X c-commands a constituent Y if, and only if,

(a) X's sister is Y, or

(b) X's sister contains Y

Take (ii), for example:

(ii)
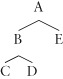

C's sister neither is E nor contains E, so C does not c-command E. E's sister however is B and B contains C, so E c-commands C.

There are structural conditions on recovering this kind of meaning from sentences, and again c-command seems to be relevant. One important view takes the generalization to be as follows:

(40) The expression which supplies the meaning to a reflexive c-commands the reflexive.

It then correctly follows that (41) cannot mean that Lilly is such that her father impresses Lilly, since the word *Lilly* does not c-command the reflexive *herself*. I have placed brackets in to mark the constituent structure:

(41) *[ [ [ Lilly's ] father ] [ impresses herself ] ]

In this example, the phrase that c-commands *herself* is *Lilly's father*, and since that phrase is grammatically masculine, and *herself* is grammatically feminine, no bound variable interpretation is possible, leading to unacceptability.^82^ In contrast, in *Lilly impresses herself*, the subject *Lilly* c-commands the reflexive, and so can supply the relevant meaning.

Another case where we find a bound variable semantics is resumptive pronouns. These are found in many language families (Celtic, Semitic, Kru, Germanic, Polynesian), and appear in structures that look as follows (the following is Irish, from McCloskey,^83^ example 10) with the rough literal translation given:

(42) Céacu fear ar labhair tú leis

Which man that talked you to-him?

*literally*: ‘Which man did you talk to him?’

The meaning of this question is roughly: *for which x, where x is a man, is it the case that you talked to x*. The pronoun *him* and the constituent *which man* are construed together, giving us a bound variable semantics. McCloskey (2006) provides a survey of the empirical range of resumptive pronoun structures and their theoretical analysis.

The equivalent of (42) in English is of course (43a), with further examples of the construction in (b-c):

(43)**a.** Which man did you talk to?
**b.** Which girl did the cat scratch?
**c.** What scratched the girl?

These also have a bound variable reading, but there is no pronoun or other overt element to mark the position of the variable. Instead there is simply the absence of any phrase, often termed a *gap*. This same pattern appears in many distinct places in the grammar of English and other languages. (44) shows it also in relative clauses and topicalizations, where there is a gap where the object of the verb *catch* would usually be (a much wider range of cases can be given here^84^):

(44)**a.** The mouse that the cat caught died.
**b.** That mouse, the cat will never catch.

One possible analysis is that there is an unpronounced pronoun in the position of the object, so that English works just like a language with resumptive pronouns. However, both linguistic and experimental evidence has shown that gaps in English do not behave like resumptives either linguistically^83^,^85^ or experimentally.^86^,^87^

The construction exemplified in (43) is called a *constituent question*, since the constituent at its beginning is the focus of the question. This construction has spawned a vast literature because of the linguistic and processing issues that it raises. It is found in matrix clauses and also in embedded clauses:

(45) Guess which girl the cat scratched!

In many languages, the constituent that is questioned is specially marked; in English the relevant marking often involves words containing *wh*, hence the other name for these questions: *wh*-questions.

Recall the generalization (32), that requires a subject to appear in the specifier of T, holds in English. A similar generalization holds in English for constituent questions, presented here in a somewhat simplified form:

(46) In a constituent question in English, a wh-constituent has to appear in the specifier of C.

Evidence for this comes from the ungrammaticality of examples like the following where there is no wh-constituent to the left of the C position and the object *which girl* is in the normal object position, sister to the verb (compare with example (43-b) and (45), respectively):

(47)**a.** *Did the cat scratch which girl?
**b.** *Guess the cat scratched which girl!

The tree for (45) is then:

(48)
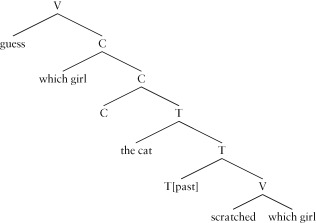


Here *scratch* subcategorizes for an object, but in this case the speaker has chosen to ask a question about that object, so it is marked as a wh-phrase, and the generalization in (46) requires it to appear in the specifier of C, hence it is pronounced to the left of the sentence. Just as we saw with the structural subject position in English, the relevant constituent enters into two distinct positional dependencies (each with an associated semantics in this case), and only the higher of the two constituents is pronounced, giving a movement/displacement phenomenon.

The generalization about wh-constituents does not, however, apply to all languages. Many languages (e.g. Japanese, Chinese) leave the *wh* constituent in its position inside the sentence, so we have sentences like the following in Japanese, where the wh-constituent remains in the same position it would be in if it were just a simple object (to the left of, and sister to, the verb):

(49) Taroo-wa nani-o kaimasita ka?

Taro what bought Q

‘What did Taro buy?’

Here the complementizer that marks the sentence as a question is at the end of the sentence, and is marked just with a Q in the gloss: but the hierarchy is the same as English, although the order is partially reversed, and the wh-constituent stays in the object position. Japanese is said to *set two syntactic parameters* differently from English: (i) one parameter is the order of the complements of C, T and V, and (ii) the other is the lack of a requirement that the specifier of C is filled in a wh-question:

(50)
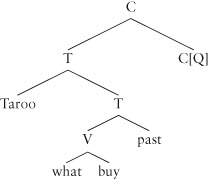


One final point about bound variable dependencies is important. Although they are structurally restricted, they appear to be unbounded:

(51) Which problem did you intuit [ that Anson would say [ his administrator would persuade [ Morag to solve ] ] ]?

Here the gap to be associated with *which problem* is separated from it by three clauses (marked in the example with brackets). Although memory issues quickly intervene, no one has ever established a systematic limit on how many sentence boundaries this *wh*-dependency can cross, and the class of dependencies that work this way are often referred to as unbounded dependencies (or long-distance dependencies). However, there is linguistic evidence that the C position after each verb c-commanded by the *wh*-phrase (in this case, *intuit*, *say*, *persuade*, *solve*) is part of the dependency^88^–^90^ and experimental evidence that the human sentence processor posits a gap in these positions.^91^ This has been taken as evidence that apparently long-distance dependencies like this are composed of a series of shorter dependencies strung together.^92^

Connected to this issue, even long distance dependencies are subject to certain locality effects^93^ known as Island Conditions. For example, although a *wh*-constituent may link to its gap over intervening sentences, it may not if the intervening sentence is itself a subject, or is inside a noun phrase. In (52), *that Lilly scratched Anson* is the subject of the verb *surprise*, but it is impossible to make a constituent question where the object of *scratch* appears as the specifier of C; in (53), the object *the mouse* contains a modifying sentence (a relative clause) which itself contains a further constituent *Anson*. Again, this constituent cannot be syntactically questioned:

(52)**a.** [ That Lilly scratched Anson ] surprised David.
**b.** *Who did that Lilly scratched surprise David

(53)**a.** Lilly caught [ the mouse that Jasper brought Anson ]
**b.** *Who did Lilly catch the mouse that Jasper brought.

There are many Island configurations, and an enormous amount of work has gone into understanding their nature, their cross-linguistic validity, and their explanation.^94^–^96^ Whether they are to be understood as emerging from the structure of the grammar^92^ or from the structure of other cognitive processes (such as the sentence processor^97^) is a current topic of debate^98^), but the empirical phenomenon is robust and clearly important to cognitive science as a whole.

This section has sketched only a very few of the most important kinds of syntactic dependencies found in human language. Others involve obligatory interpretation of the arguments of different predicates as the same entity, as in (54a), where the understood subject of *catch* is interpreted as identical to the subject of *attempt*; apparent anti-dependencies where a pronoun and a noun phrase cannot be construed as the same (as *she* and *Lilly* in (54b)); and cases where there is a single *wh* constituent, but multiple gaps, as in (54c), where *which mouse* is understood as the object of both *scratch* and *ate*. Of course, these are just examples from English, while research in syntax investigates how or if they appear in other languages and what consequences the variation and uniformity across languages has for understanding the nature of the human syntactic capacity.

(54)**a.** Lilly attempted to catch the mouse.
**b.** She didn't understand that Lilly was sleepy.
**c.** Which mouse did Lilly scratch before she ate?

## CONCLUSIONS

We began by divorcing syntax from the more general functions of language, but it is clear that syntactic research can shed some light on these more general questions. Structure, after all, both enhances and constrains function. The system that generates constituent structures enriches human cognitive and communicative abilities by providing us with an unbounded range of structures connecting sound with meaning and helping to explain why humans outstrip other species in this domain; discoveries about the positions and meanings of functional categories across languages and how these categories influence the shape and meanings of syntactic structures provide evidence for how language relates to more general categories of thought, such as agency, time, and objecthood; the discovery that units of syntax may enter into a number of dependencies simultaneously and how these dependencies are realized in different ways in different languages has provided new ways of thinking about what is universal versus what is variable in human cognition and hence what is subject to change over generations. Decades of syntactic research have uncovered not only a huge wealth of knowledge about how human language works in the domain of syntax, but have also provided cognitive science with theoretical models of this apparently unique aspect of human nature.

## NOTES

a When I present material from languages other than English, I will usually suppress technical information in the gloss, on the assumption that most readers will be interested in the patterns rather than the specifics of the data. For those interested, the references provide the source and example numbers of the data.

b Noun classes in Tsez are only roughly semantically determined, with class I being male humans, class II being female humans, plus a number of inanimate nouns, class III including all animals and a large set of inanimates, and class IV being inanimate nouns.
